# How Brain Waves Help Us Make Sense of Speech

**DOI:** 10.1371/journal.pbio.1001753

**Published:** 2013-12-31

**Authors:** Janelle Weaver

**Affiliations:** Freelance Science Writer, Carbondale, Colorado, United States of America; PLOS, United Kingdom

Understanding a sentence that someone else utters may seem effortless to you, but it's actually a complex process because you have to parse that sentence into many parts to understand what it means. Speech consists of a hierarchy of components that each takes place on a different timescale. Speech cues such as intonation occur on a relatively long timescale, unfolding over hundreds of milliseconds. At the other end of the spectrum is the phoneme—the smallest unit of speech—which lasts only tens of milliseconds.

**Figure:**
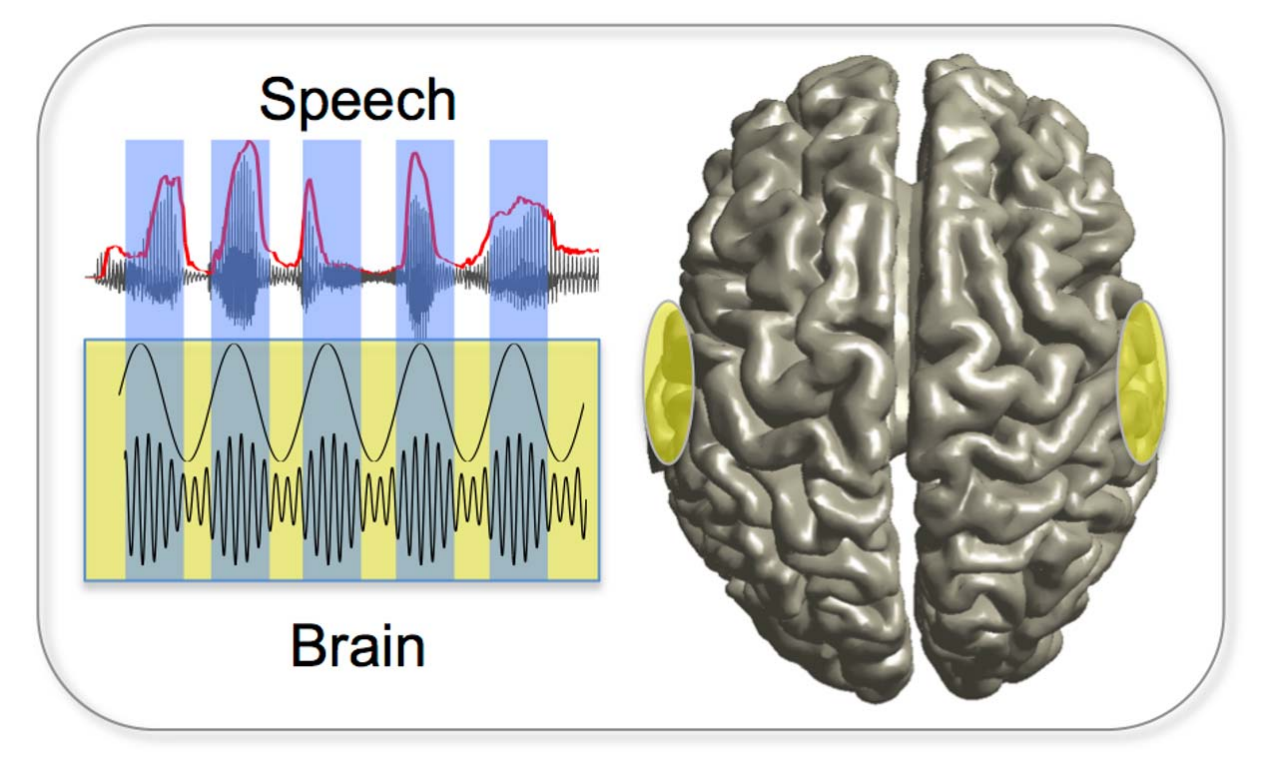
Gross and co-workers show that hierarchical speech patterns (top) entrain hierarchical wave frequencies in the brain (bottom).

Similarly, distinct sets of neurons in the brain fire rhythmically at different rates, and these oscillations can also be arranged in a hierarchy. For instance, slow delta-wave oscillations can influence the magnitude of faster theta oscillations, which in turn can alter the amplitude of even faster gamma oscillations. Rhythmic brain activity plays an important role in a variety of cognitive processes, including attention, memory, and decision-making.

A new study published in *PLOS Biology* now offers insights into the important role that neural oscillations play in speech perception. Joachim Gross, Simon Garrod, and co-workers report novel evidence that hierarchically organized neural oscillations allow people to parse the hierarchical components of speech. The findings not only provide a more complete understanding of the role of brain oscillations in cognition, but they also reveal that people can efficiently understand speech by simultaneously processing different speech components on various timescales.

Gross and his team used a brain imaging technique called magnetoencephalography to record neural activity in 22 volunteers while they listened to a seven-minute real-life story. The researchers found that neural oscillations were arranged in a hierarchy: delta oscillations influenced the magnitude of theta oscillations, which in turn affected the amplitude of gamma oscillations.

These hierarchical oscillations matched the hierarchical components of speech. Slow delta-wave oscillations in brain regions that process auditory information were in sync with slow rhythmic changes in speech, corresponding to the timescale of prosody—the rhythm, stress, and intonation of speech. This temporal alignment was more pronounced in the right hemisphere of the brain than in the left hemisphere.

Meanwhile, the magnitude of fast gamma-wave oscillations in auditory brain areas was influenced by changes in speech that took place on a relatively short timescale, corresponding to syllables. This effect was stronger in the left hemisphere than in the right hemisphere. Collectively, these findings are consistent with a theory called “asymmetric sampling in time” (AST), which proposes that the right hemisphere preferentially processes information over longer timescales, whereas the left hemisphere shows a bias for processing information over shorter timescales.

Gross and his collaborators then examined how brain oscillations were affected by variations in speech over time. Speech is not strictly periodic; sometimes people unexpectedly stop talking or begin to talk more quickly. The researchers found that sudden, large changes in the physical features of speech reset the timing of oscillations in auditory brain areas, helping to sync up important speech events with oscillatory brain activity. This finding illustrates how oscillations adapt to changes in the rhythm of speech, allowing the brain to continue to efficiently process speech even when the physical features abruptly change.

Taken together, the findings show that hierarchically organized brain oscillations work in concert to track speech components occurring at different timescales, helping to convert a continuous speech stream into meaningful internal representations.

**Gross J, Hoogenboom N, Thut G, Schyns P, Panzeri S, et al. (2013) Speech Rhythms and Multiplexed Oscillatory Sensory Coding in the Human Brain.** doi:10.1371/journal.pbio.1001752

